# Preliminary psychometric properties of the Chinese version of the structured interview of personality organization (STIPO-CH)

**DOI:** 10.1186/s12888-023-05041-y

**Published:** 2023-08-07

**Authors:** Yang Wang, Zirong Li, Jie Zhong

**Affiliations:** 1 Beijing Key Laboratory of Behavior and Mental Health Clinical and Health Psychology Department,School of Psychological and Cognitive Science, Peking University, 5 Yiheyuan Road, Beijing, 100871 China; 2 Civil Aviation Medicine Center of Civil Aviation Administration of China, Beijing, China

**Keywords:** Personality organization, Structured interview, Reliability, Validity, Personality disorder

## Abstract

**Background:**

Kernberg originally proposed the psychoanalytic concept of personality organization (PO), which measures personality pathology from a dimensional approach with multiple scales and can be evaluated using the Structured Interview of Personality Organization (STIPO) from six domains: identity, object relations, primitive defenses, coping vs. rigidity, aggression, and moral values. The present study translated the original version into the Chinese STIPO (STIPO-CH) version and evaluated its reliability and validity.

**Methods:**

The STIPO-CH was administered to 49 non-clinical subjects. They also completed the Chinese version of the Inventory of Personality Organization and the Millon Clinical Multiaxial Inventory to evaluate criterion-related reliability. Interrater reliability was assessed with intraclass correlations. An item analysis was carried out to explore the structure and internal consistency.

**Results:**

Interrater reliability (intraclass correlations) ranged from 0.98 to 0.99. Results suggested acceptable internal consistency for identity and moral values. The correlations between STIPO-CH domains and self-report questionnaires indicated that construct validity and criterion-related validity were acceptable to good.

**Conclusions:**

Overall, this study presents preliminary psychometric properties of STIPO-CH. Limitations regarding the sample, interviewers, and cultural differences are discussed. Future research is highly recommended.

## Background

Personality organization is a psychoanalytic concept proposed by Otto Kernberg [1], which describes personality from a dimensional perspective [2]. This framework, derived from contemporary object relations theory, proposes that individuals’ personality functioning is organized by internal stable structures. Kernberg’s [1, 3] diagnostic and theoretical framework suggest that the development of personality disorders (PDs) is typically associated with impairment in PO due to a range of neurophysiological (e.g., temperament type, aggression) and environmental (e.g., trauma, neglect, etc.) factors. As an alternative to the traditional categorical system of PDs, PO demonstrates significant potential in research and clinical practice. Instruments assessing PO can be used to explore the psychopathology of general impairment in personality functioning rather than symptoms. Besides, as a psychoanalytic concept, clinicians practicing psychoanalysis or psychodynamics can utilize PO to evaluate therapeutic effectiveness. Therefore, cross-cultural validation is highly in need.

In the original model, the levels of PO are measured through three main dimensions: identity, defense mechanisms, and reality testing. The identity dimension refers to the existence of a cohesive, integrated, and relevantly stable representation of the self and others, which determines the ability to integrate positive and negative traits of the self and others [4, 5]. The dimension of primitive defense evaluates individuals’ emotional, cognitive, and behavioral experiences of using primitive defense mechanisms [6]. Furthermore, the reality testing dimension refers to whether individuals can distinguish between experiences from the internal and external worlds.

In addition to these three main dimensions, Kernberg, Clarkin, Caligor, and Stern [7] also identify four more dimensions of PO, including the quality of object relations, aggression, coping strategies, and moral values. The quality of object relations consists of interpersonal functioning and intrapsychic feelings toward themselves and others during social interactions. Aggression is assessed through the level to which psychological status and behaviors are determined by aggressiveness or defense mechanisms against it. Coping strategies refer to how individuals respond to stressors, whether they can stay resilient and adaptive or remain automated and fixed. The dimension of moral values contains inner values and morals that direct intrapsychic experience and behavior.

Based on the continuum of severity, personality pathology is categorized into three levels of PO, from mild to severe, respectively: neurotic level, borderline level, and psychotic level. A neurotic level of PO represents intact reality testing, the use of mature defense mechanisms (e.g., repression) and integrated identity. Borderline PO, on the other hand, is characterized by impaired reality testing, primitive and immature defenses, unstable relationships, aggression towards the self or others, and identity diffusion. Individuals at the psychotic level of PO have severe deteriorations in all PO dimensions. According to their clinical experience, Kernberg and colleagues [2] associated each DSM-characterized PD with different levels of severity of PO. Since the range of PO for each PD varies (e.g., narcissist PD ranges from psychotic to neurotic PO, while obsessive-compulsive PD only locates at neurotic PO), evaluating PO in addition to diagnosing PD is highly beneficial for case conceptualization and treatment planning.

Previous research has vastly supported the applicability of PO by demonstrating the relationship between the level of PO and various clinical conditions. For example, Vermote et al. [8] found that a lower level of PO was correlated with more severe symptoms of self-injury, anxiety, depression, and intense anger in psychiatric inpatients. Other studies focusing on PDs reported that identity diffusion and primitive defenses were significantly correlated with borderline and paranoid personality features, while lower reality testing was correlated with higher scores on borderline and schizotypal personality [9]. It was also suggested that the level of reality testing could predict dissociative symptoms in both clinical and non-clinical populations [10]. Thus, as most data were collected in clinical settings, more research is needed to investigate the relationship between PO and psychological features in community samples.

## Development of the structured interview for personality organization (STIPO)

The Structured Interview for Personality Organization (STIPO; 4) was developed for comprehensive examinations in research and clinical practice with higher reliability and validity than the original version of the structural interview [11]. Interviewers rate each of the 87 questions on a 3-point scale, with 0 indicating no pathology and 2 indicating a clear presence of pathology. The domain scores can be calculated by averaging the items with 0–2 scores or on a 1–5 scale based on interviewers’ clinical judgment. Key components of the PO are examined in terms of behavior and cognition, including identity stability, object relations quality, use of primitive defense mechanisms, aggression, adaptive coping versus rigidity, and moral values. Identity stability is assessed by asking about commitment to work or schoolwork and recreation, feelings about self (e.g., knowledge and understanding of self and self-esteem), and perceptions of others (e.g., the accuracy of interpersonal perceptions and the knowledge and understanding of significant others). Questions examining the quality of object relations inquire about participants’ existence and quality of closed friendship and intimate relationships, the combination of love and sex, care and empathy for significant others, and the stability of the above factors. Regarding primitive defense, STIPO focuses on the situation, frequency, beliefs, and attitudes about paranoia, eccentric behavior, idealization/deprecation, and primitive denial. The section on coping and rigidity evaluates how individuals react to stressful situations. Questions about aggressiveness measure the extent to which an individual’s internal cognition and external behavior are dominated by aggression or defenses against aggression. Finally, the sections on moral value collect information about intrinsic standards and the extent to which they influence decision-making.

Existing studies examining the psychometric properties of STIPO in other languages have yielded satisfactory results. Doering et al. [12] applied the German version of STIPO and reported that Cronbach’s *α* ranges from 0.69 to 0.93, while intraclass correlations range from 0.89 to 1, demonstrating good internal consistency and inter-rater reliability. Patients diagnosed with PD generally scored higher on STIPO than healthy controls, while those diagnosed with clustered B PD also scored lower than those with cluster C PD. The Italian version also reported high inter-rater reliability with intra-class correlations (ICCs) ranging from 0.82 to 0.97 [13]. However, since most of the literature was conducted in clinical settings, the potential of STIPO in community samples remains unknown.

Notably, a revised version of STIPO (STIPO-R; [14, 15]) was recently developed in response to the need for a more efficient instrument to evaluate PO. Essential items from STIPO were selected and modified, resulting in a final pool of 55 items that covered five main domains (i.e., identity, object relations, defenses, aggression, and moral values) and an additional embedded domain of narcissism. Same as STIPO, STIPO-R operates two scoring systems for domains: (1) the average of the 0-1-2 scores of all items; (2) an overall clinical rating ranging from 1 to 5. A notable update of STIPO-R is a reference table that links the clinical ratings of the five domains to six levels of PO (i.e., normal PO, neuritic PO 1, neurotic PO 2, high-level BPO, middle-level BPO, and low-level BPO). For example, a score of 1 at all domains signifies a normal PO, while a score of 5 (range 4–5) at object relations, and 4 or 5 at other domains represents a low-level BPO. However, since the reliability and validity of STIPO-R are still under evaluation, this study intends to translate and evaluate the original version of STIPO.

As either STIPO or STIPO-R are long-term interviews that require the administration of a well-trained professional, self-report questionnaires can be complimentary screening tools. The Inventory of Personality Organization (IPO; [16]) is an 83-item self-report inventory that includes three major dimensions (i.e., identity diffusion, primitive defenses, and reality testing) and two additional dimensions (i.e., aggression and moral values). Researchers also developed shorter versions of the IPO with 30 [17] or 18 items [18], the psychometric properties of which require further validation though. The Chinese version of IPO (IPO-CH; [19]) contains 48 items and demonstrates good reliability (Cronbach’s α ranges from 0.78 to 0.93).

## Relationship between STIPO and recent changes in diagnosing PD

In contrast to the categorical system outlined in the DSM-5, which relies on symptomatology to assess personality pathology, PO provides a detailed assessment of personality development. Meanwhile, the traditional classification of PDs has been continuously challenged by researchers due to the high comorbidity between PDs, difficulty in assessing severity, and lack of empirical support for diagnostic thresholds [20, 21]. In this case, the Alternative Model for PDs (AMPD) is proposed in Section III of the DSM-5, which aims to diagnose PDs through impairment in personality functioning and nonadaptive personality traits [22]. Personality functioning consists of four dimensions categorized into two types: self-functioning, which includes identity and self-direction, and interpersonal functioning, which includes empathy and intimacy [23]. According to Hörz-Sagstetter et al.’s [24] review, PO and personality functioning share a common factor, and this convergence in theoretical conceptualization has been supported by empirical evidence. For example, in a study that administered STIPO and the Structured Interview for DSM–5 Alternative Model for Personality Disorder Module I (SCID-AMPD; [25]), total and dimensional scores of the interviews significantly correlated, while both interviews demonstrated high correlations with clinical relevance of personality pathology such as suicidal attempt and psychiatric hospitalization [26]. Clinician-rated scores of three dimensions of personality functioning (i.e., identity, self-direction, and empathy) by watching recordings of STIPO could also differentiate whether interviewees have diagnosis of personality disorders, diagnosis of other mental illness, or are healthy controls [27].

Another major update in diagnosing PDs comes with the publication of the International Classification of Diseases, 11th Edition (ICD-11; [28]), which radically switches the categorical diagnosis of PDs into a dimensional classification. This diagnostic system involves a two-step evaluation: severity (personality difficulty, mild PD, moderate PD, and severe PD) and prominent traits (negative affectivity, detachment, dissociality, disinhibition, and anankastia; [29]). The measurement of severity shares overlaps with the operationalization of STIPO, especially in the dimensions of identity and reality testing [30, 31].

## The current study

As a reliable and clinically valid measurement for PO, STIPO can be used to evaluate the severity of personality pathology from a dimensional approach, inform the intensity and type of treatments, and provide insights into the development of diagnostic models for PDs [4, 7, 12]. In this case, it is necessary to explore the applicability of STIPO with a culturally diverse population. The current study aims to fulfill this need by translating STIPO into the Chinese version (STIPO-CH) and preliminarily evaluating its psychometric properties. As a pilot study, the current research recruited a community sample for convenience. Since most existing studies (e.g., 4,12) administered STIPO to patients with clinical diagnoses, and because several Chinese clinicians considered the diagnosis of PD as untreatable and meaningless [32], this research examined the psychometric properties of STIPO in non-clinical populations. Two questionnaires that are validated in the Chinese population and conform to the categorical and dimensional approach of conceptualizing personality pathology served as external criteria to evaluate criterion-related validity. It is generally expected that STIPO-CH would demonstrate acceptable to good reliability, construct validity, and criterion-related validity. Specifically, domains of STIPO-CH were hypothesized to significantly and positively correlate with their self-report parallels. According to Kernberg’s [3] proposition in the connections between PO and DSM-classified PDs, researchers anticipated significant associations between identity and primitive defenses and Cluster B PDs (e.g., borderline, histrionic) and negative affect, moral values with antisocial personality and substance use.

## Methods

### Participants

Fifty-two participants were recruited online in the Beijing area, and three were excluded for failing to complete the questionnaires, resulting in a final sample size of 49. Of the participants, 19 (38.78%) were male, 30 (61.22%) were female, and ages ranged from 20 to 52 (*M* = 28.0, *SD* = 7.8). Fifteen (30.61%) reported a married status. Twenty-nine (59.18%) of the participants were students in university settings, while the other 20 (40.82%) had worked for at least three years.

### Materials

#### The Chinese version of the structured interview of personality organization (STIPO-CH)

The STIPO [4] consists of 87 questions to assess the PO level through structured interview, which takes about 90 to 180 min. Each question is accompanied by one or more follow-up questions, which the interviewer is required to read to the participant and, if needed, dig further in depth. Six domains are assessed: identity, object relations, primitive defenses, coping vs. rigidity, aggression, and moral values. The interviewer assigns three points to each question: 0 indicates that the symptom is not present or has no impact on functioning, 1 indicates that the symptom is present with a minor impact on functioning, and 2 indicates that the symptom is present and causes severe impairment of functioning. The STIPO user manual includes two algorithms for scoring domains and subdomains: [1] calculate a mean value for all items in the particular section; and [2] use a 5-point Likert scale (1 = good functioning; 5 = severely impaired functioning) for the overall section. A higher score represents more severe pathology. The first author of this study conducted the translation after obtaining permission from the original author, and this draft was back-translated by other research members. The authors then inquired about specific wording from the original author. Through repeated discussions with the research team, the final version was generated under the guidance of a registered psychologist/supervisor in the Chinese Psychology Society.

#### The millon clinical multiaxial inventory-III (MCMI-III)

The MCMI-III [33] is chosen as a criterion because it is a comprehensive and useful tool that assesses personality pathology and clinical syndromes with verification questions. This 175-item instrument consists of 29 scales and anticipates yes-or-no answers. There are five correction indexes: one validity index that assesses whether participants answer items randomly, and three modifying indexes that signifies biased response pattern including being self-revealing or secretive, inclining to appear socially desirable, or disproportionally devaluing oneself. The 24 clinical indexes include 3 severe personality disorders (i.e., borderline, paranoid, and schizotypal), 11 personality pathology, 3 severe clinical symptoms (i.e., thought disorder, major depression, and delusional disorder), and 7 other clinical symptoms (see Table 1). The Chinese version was translated and revised by Li et al. [34], which demonstrates good reliability (Cronbach’s *α* = 0.96, split-half reliability = 0.92, and test-retest reliability = 0.71).

**Table 1:** Correlations between STIPO-CH Domains and Questionnaires

	Identity	Object Relations	Primitive Defenses	Coping vs. Rigidity	Aggression	Moral Values
IPO-CH						
Primitive Defenses + Identity Diffusion	0.37**	− 0.03	0.20	0.28	− 0.11	0.05
Reality Testing	0.16	0.09	0.09	− 0.18	0.14	0.24
Aggression	0.24	− 0.10	− 0.05	− 0.14	0.22	0.32*
Moral Values	0.18	0.11	0.04	0.03	0.00	0.29*
MCMI-III						
Disclosure	0.12	− 0.09	0.14	0.01	0.02	− 0.02
Desirability	− 0.40**	− 0.09	0.03	− 0.28*	0.15	− 0.09
Debasement	0.11	− 0.03	0.12	0.13	− 0.12	0.02
Schizoid	0.28	0.25	− 0.17	− 0.02	− 0.05	− 0.10
Avoidant	0.31*	0.23	0.00	0.26	− 0.07	− 0.18
Depressive	0.19	− 0.08	0.29*	0.22	− 0.04	− 0.02
Dependent	0.21	− 0.11	0.33*	0.20	− 0.18	− 0.12
Histrionic	− 0.32*	− 0.20	0.32*	− 0.31*	− 0.06	− 0.06
Narcissistic	− 0.27	− 0.23	− 0.06	− 0.38**	0.12	0.11
Antisocial	0.02	− 0.05	− 0.02	0.03	0.23	0.28
Aggressive	− 0.07	− 0.20	− 0.05	− 0.01	0.34*	0.21
Compulsive	− 0.13	0.15	− 0.07	− 0.12	− 0.22	− 0.11
Passive-Aggression	0.11	− 0.18	0.24	0.13	− 0.04	0.09
Self-defeating	0.23	0.16	0.00	0.12	− 0.07	− 0.14
Schizotypal	− 0.05	0.02	− 0.03	− 0.12	0.09	0.30*
Borderline	0.20	− 0.12	0.23	0.19	− 0.04	0.11
Paranoid	0.12	− 0.16	− 0.12	− 0.11	0.20	0.28
Anxiety	0.22	− 0.06	0.13	− 0.02	− 0.15	− 0.14
Somatoform	0.30*	− 0.16	0.00	0.10	− 0.02	0.19
Bipolar-manic	− 0.01	0.07	0.36*	0.04	0.06	0.06
Dysthymia	0.33*	0.00	0.11	0.17	− 0.19	0.06
Alcohol dependence	− 0.05	− 0.15	− 0.15	− 0.16	0.11	0.11
Drug dependence	0.13	− 0.06	0.12	0.02	0.16	0.32*
PTSD	0.13	− 0.10	0.20	0.12	0.03	0.10
Thought disorder	0.09	− 0.04	− 0.02	0.07	0.03	0.03
Major depression	0.32*	− 0.18	− 0.03	0.13	− 0.02	0.16
Delusional disorder	0.01	− 0.05	− 0.04	− 0.22	0.18	0.24

*Note*: *p < .05; **p < .01

#### The Chinese version of inventory of personality organization (IPO-CH)

The IPO-CH [19] is a self-report questionnaire that quantifies personality organization. As a parallel measure of STIPO that derives from the same concept, it has been used for the construct validation of STIPO [4]. It comprises of 48-loading on four subscales: primitive defenses + identity diffusion, reality testing, aggression, and moral values. All items were rated using a 5-point Likert scale: 1 = never, 2 = rarely, 3 = sometimes, 4 = often, and 5 = always. Subjects were asked to read each item and then select a number that best matched their daily activities, feelings, thoughts, and relationships. Cronbach’s *α* ranges from 0.78 to 0.93 for the dimensions, and test-retest reliability = 0.60.

### Procedure

After signing the informed consent form, participants were first interviewed about their life history. This non-structured interview lasted 60 min and was aimed at forming an alliance between the interviewer and the respondent and obscuring the research goal. Then, self-report questionnaires were administered. The STIPO-CH interviews were carried out by five clinical psychology students or professionals who had received training in general psychoanalysis, personality assessment, Kernberg’s theory of PO, and the use and algorithms of STIPO-CH. Each interviewer conducted a pilot assessment, the materials and scoring of which were discussed together under supervision. Training and supervision were delivered by an experienced clinical psychologist, who is a member of the International Psychoanalytic Association, a candidate of the International Society for the Study of Personality Disorders, and a registered supervisor of the Chinese Psychological Society. Two months following the interview, each participant received souvenirs worth 50 Chinese Yuan and feedback. Peking University granted ethical approval (Project Number: 2020-04-27), which approved all experimental protocols, and all methods were performed in accordance with the guidelines and regulations.

### Analysis

Intra-class correlations were calculated to assess inter-rater reliability. Considering the influence of the interviewer as a factor on the scoring results, five interviews were randomly selected from the total interview material and independently scored by two interviewers based on the recordings, thus using the ICC to compare the evaluator consistency coefficients. Cronbach’s α was calculated for each STIPO-CH dimension to determine the internal consistency. The construct validity of STIPO-CH was evaluated by calculating correlations between subdomains and domains of STIPO-CH, while correlations between dimensions of STIPO-CH, IPO-CH, and MCMI-III were calculated to evaluate the criterion-related validity. SPSS 17.0 was used for statistical analysis in this study. The 2-point Likert scale was used for calculating inter-rater reliability, item correlation, and internal consistency, while the 5-point arithmetic scale was applied for examining construct validity and criterion-related validity.

## Results

The correlation between the two algorithms (arithmetic and five-point scale) equals 0.92 for identity, 0.84 for object relations, 0.78 for primitive defenses, 0.90 for coping vs. rigidity, 0.76 for aggression, and 0.96 for moral values. All correlations were significant at the 0.05 level, indicating high consistency between the two algorithms.

### Inter-rater reliability

Five participants were randomly selected for a second scoring, which covered two interviewers. The intra-class correlations ranged from 0.98 to 0.99 (*M* = 0.99), indicating good inter-rater reliability.

### Item analysis and internal consistency

For each question in Table 2, the mean, standard deviation, and correlation with the belonged domain were calculated. Internal consistency was acceptable for identity (Cronbach’s *α* = 0.73) and moral values (Cronbach’s *α* = 0.79), questionable for object relations (Cronbach’s *α* = 0.65), poor for coping vs. rigidity (Cronbach’s *α* = 0.52), and unacceptable for primitive defenses (Cronbach’s *α* = 0.19) and aggression (Cronbach’s *α* = 0.29).

**Table 2:** Descriptive Statistics for Items and Domains

	*M*	*SD*	Correlation with Domain
Identity	1.8	0.77	
1/5 Work/Studies Effectiveness	0.30	0.49	0.35*
2/6 Work/Studies Stability	0.17	0.43	0.06
3/8 Work/Studies Ambition/Goals	0.58	0.64	0.35*
4/7 Work/Studies-Satisfaction	0.52	0.57	0.27
9 Presence of Sustained Interests	0.43	0.54	0.40**
10 Picks Up and Drops	0.41	0.50	0.39**
11 Satisfaction	0.41	0.50	0.28
12 Sense of Self	0.37	0.57	0.64**
13 Ambivalence	0.35	0.56	0.49**
14 Reflective Functioning	0.59	0.71	0.58**
15 Consistency across Time	0.08	0.45	0.29*
16 Tastes/Opinions	0.25	0.52	0.15
17 Consistent Sense of Self in Present	0.29	0.58	0.05
18 Time Alone	0.16	0.37	0.41**
19 In Intimate Relationships	0.33	0.53	0.01
20 Self Esteem	0.55	0.68	0.08
21 Representation of Others	0.43	0.54	0.45**
22 Ambivalence	0.47	0.68	0.49**
23 Reflective Functioning	1.0	0.74	0.54**
24 Assessing Others	0.63	0.64	0.30*
25 Other’s Judgments	0.33	0.63	0.44**
26 Social Reality Testing	0.45	0.50	0.07
27 Description Family of Origin	0.33	0.59	0.41**
28 Ambivalence	0.39	0.61	0.36*
29 Reflective Functioning	0.94	0.72	0.52**
Object Relations	1.4	0.50	
30 Close Friendships	0.10	0.31	0.26
31 Depth of Close Friendships	0.23	0.42	0.11
32 Problems/Volatility	0.06	0.24	0.11
33 Friends’ Unavailability	0.06	0.24	0.10
34 Temporal Stability	0.27	0.45	0.25
35 Intimacy/Interdependency	0.53	0.65	0.59**
36 Conflict/Volatility	0.72	0.70	0.28
37 Capacity for Investment	0.74	0.86	0.61**
38 Need Fulfilling	0.19	0.40	− 0.04
39 Bored	0.19	0.40	0.34*
40 Do Better	0.36	0.49	0.39*
41 Critical	0.31	0.53	0.54**
42 Sexual Activity	0.39	0.57	0.65**
43 Sexual Inhibit	0.11	0.32	0.29
44 Sensual Pleasure in Sex	0.76	0.86	0.52**
45 Love and Sex	0.21	0.50	0.59**
46 Concern for Other	0.12	0.33	0.38**
47 Envy	0.31	0.47	0.24
48 Entitlement	0.65	0.48	0.29*
49 Autonomy of Other	0.45	0.50	− 0.05
50 Need Fulfilling 1	0.25	0.48	0.07
51 Need Fulfilling 11	0.31	0.47	0.11
Primitive Defenses	1.2	0.37	
52 Paranoia	0.35	0.56	0.29*
53 Erratic Behavior	0.29	0.50	0.32*
54 Idealization/Devaluation I	0.31	0.55	0.51**
55 Idealization/Devaluation II	0.14	0.41	0.55**
56 Primitive Denial	0.16	0.37	0.15
57 Projective Identification	0.20	0.41	0.35*
58 Fantasy	0.12	0.33	0.31*
59 Somatization	0.41	0.54	0.41**
60 Over-Reaction	0.27	0.49	0.36*
Coping vs. Rigidity	1.4	0.64	
61 Anticipation/Planning	0.37	0.67	0.61**
62 Suppression	0.63	0.70	0.59**
63 Flexibility	0.25	0.43	0.52**
64 Stress Response	0.49	0.62	0.63**
65 Self Blame	0.23	0.51	0.30*
66 Control I	0.47	0.54	0.19
67 Control II	0.27	0.57	0.54**
68 Challenges	0.39	0.49	0.45**
Aggression	1.1	0.28	
69 Self Neglect	0.20	0.46	0.36*
70 Risky Behavior	0.27	0.45	0.08
71 Self-Injury	0.10	0.31	0.26
72 Suicidality	0.02	0.14	0.14
73 Sexual Aggression – Self	0.00	0.00	-
74 Temper	0.59	0.61	0.61**
75 Attacks on Others	0.10	0.37	0.45**
76 Enjoyment of Suffering of Others	0.14	0.35	0.50**
77 Sexual Aggression - Others	0.00	0.00	-
78 Intimidation	0.31	0.51	0.64**
79 Revenge	0.33	0.52	0.57**
Moral Values	2.1	0.96	
80 Internalized Moral Values	0.84	0.72	0.72**
81 Deceit	0.71	0.61	0.62**
82 Moral Struggle	0.98	0.69	0.66**
83 Lying	0.47	0.62	0.58**
84 Illegal Activity	0.10	0.31	0.35*
85 Guilt I	0.89	0.67	0.75**
86 Exploitation	0.65	0.48	0.53**
87 Guilt II	0.61	0.95	0.44**

*Note*: *p < .05; **p < .01

### Construct validity

Table 3 shows the correlations between the subdomains and domains of STIPO-CH. Results were significant for identity and coping vs. rigidity, moral values, and aggression. Besides, subdimensions of identity, object relations, and aggression (except Interpersonal Relationships from the domain of object relations) were significantly associated with the domain to which they belonged and less associated with other domains, ranging from 0.38 to 0.87. This supported the construct validity and discriminability for the three domains.

**Table 3:** Correlations between Domains and Subdomains in STIPO-CH

	Identity	Object Relations	Primitive Defenses	Coping vs. Rigidity	Aggression	Moral Values
Identity	1					
Capacity to Invest	0.59**	0.03	0.04	0.44**	− 0.09	0.14
Sense of Self	0.77**	0.16	− 0.19	0.15	− 0.15	0.08
Representation of Others	0.85**	0.10	− 0.32*	0.16	− 0.20	0.10
Object Relations	0.18	1				
Interpersonal Relationship	0.12	0.15	0.23	0.17	− 0.02	− 0.14
Intimate Relations and Sexuality	0.12	0.79**	− 0.29*	− 0.04	− 0.22	− 0.09
Internal Working Model of Relationships	0.21	0.38**	− 0.13	0.26	− 0.11	− 0.12
Primitive Defenses	− 0.26	− 0.21	1			
Coping vs. Rigidity	0.36*	0.13	0.04	1		
Aggression	− 0.22	− 0.22	− 0.01	0.04	1	
Self-Directed Aggression	0.21	0.38**	− 0.13	0.26	− 0.11	− 0.12
Other-Directed Aggression	− 0.02	− 0.17	− 0.26	0.07	0.45**	0.17
Moral Values	0.07	− 0.08	− 0.13	0.02	0.33*	1

*Note*: *p < .05; **p < .01

### Criterion-related validity

We calculated the correlations between the STIPO-CH, IPO-CH, and MCMI-III domains. Table 1 shows significant correlations between identity from STIPO-CH and primitive defenses + identity diffusion from IPO-CH, moral values from STIPO-CH, and aggression and moral values from IPO-CH. Most correlations between the domains of STIPO-CH and MCMI-III were significant (see Table 1).

## Discussion

The objective of the current study was to translate the Chinese version of the Structured Interview for Personality Organization (STIPO-CH), a semi-structured interview that quantifies a psychoanalytic conceptualization of personality pathology, personality organization. The psychometric properties of STIPO-CH were preliminarily evaluated. The correlations between the two scoring methods of STIPO-CH were high and significantly positive across the six dimensions, suggesting a high level of consistency. The mean value of the ICC was 0.99, with a range of 0.98 to 0.99, indicating high inter-rater reliability.

Internal consistency for identity and moral values was found to be acceptable, with values greater than 0.70. However, Cronbach’s *α* for primitive defenses, object relations, coping vs. rigidity, and aggression was lower than in previous studies (e.g., 4,12). This may be because the current study recruited non-clinical community participants through convenience sampling rather than psychiatric inpatients, thus not controlling subjects’ mental health conditions. Besides, the present sample included 49 interviewees only. As a result, some items could hardly be discriminatory. For example, in the aggression domain, all participants scored 0 and 9 for item 73 (Sexual aggression – Self) and item 77 (Sexual aggression – Others), indicating no sexual aggression in the recent five years. Thus, the psychometrics of STIPO-CH should be reexamined in a larger and more diverse sample.

Construct validity was examined by calculating correlations between domains. Significant correlations were only found between identity and coping vs. rigidity, and moral values and aggression. This finding differs from Doering et al. [12] and Stern et al. [4], which reported significant correlations for all pairs of domains. Further investigations on the subdimensions of identity, object relations, and aggression revealed that, except for Interpersonal Relationships from the domain of object relations, all other subdomains were significantly associated with the domain to which they belonged at the 0.01 level and less associated with other domains, suggesting acceptable construct validity and discriminability for these three domains.

Participants completed the MCMI-III [33] and the IPO-CH [19] to investigate the criterion-related validity of STIPO-CH. Results indicated associations between the six dimensions of STIPO-CH and different personality types and clinical conditions of MCMI-III. Specifically, being avoidant, histrionic, somatoform, dysthymic, or majorly depressed were significantly associated with higher scores on identity. As illustrated by Kernberg and Caligor [3], individuals at a higher level of borderline PO still experience identity diffusion but maintain higher functioning in relationships. Since this study recruited a community sample with a lower level of pathology, participants who scored higher on identity may present avoidant and histrionic personality rather than borderline personality. This finding was also similar to that Stern et al. [4] reported positive relationships between identity and mood problems, in alignment with Kernberg’s [1] theory that rigid internal models of self and other contribute to negative affect. Being depressed, dependent, histrionic, or bipolar-manic was significantly correlated with higher scores on primitive defense. While Stern et al. [4] also suggested a positive correlation between primitive defense and Cluster B features such as aggression and anger, Kernberg [1, 3] regards Cluster C PDs at higher levels of PO with the use of mature primitive defense. This discrepancy may be attributed to that the primitive defense domain of STIPO-CH has low internal consistency, thus unable to evaluate participants’ use of ego defense. In addition, being histrionic or narcissistic was significantly associated with lower scores on coping vs. rigidity, which was merely found in previous research. A tentative explanation may be that histrionic and narcissistic personalities, with a higher level of self-cathexis, associate with adept use of self-serving coping mechanisms. Being aggressive as measured in MCMI-III was significantly positively correlated with score on the aggression domain. Finally, levels of schizoid and drug dependence were significantly positively associated with scores of moral values. Since the dimension of moral values converges with the concept of superego, a low score on this domain may be parallel to poor self-control. However, impaired moral values are connected to antisocial and paranoid personalities in Kernberg’s [1, 3] conceptualization. It is possible that in this study, the non-clinical participants did not manifest severe psychopathy traits, and thus a low level of moral values was manifested as a lack of interest in investing or devoting to others.

Regarding IPO-CH, results indicated significant correlations between identity from STIPO-CH and primitive defenses + identity diffusion from IPO-CH, moral values from STIPO-CH, and aggression and moral values from IPO-CH, which is consistent with findings from Stern et al. [4]. However, other correlations were not significant. In contrast, Stern et al. [4] reported a significant correlation between the primitive defenses dimensions. This might be due to the limitation of self-report inventory and participants’ low self-awareness, so they may overestimate or underestimate their conditions. As participants completed self-report criteria in the current study, future research may examine the criterion validity of STIPO-CH with other interviews, such as Operationalized Psychodynamic Diagnosis (OPD-2; [35]) and Structured Clinical Interview for DSM-IV for Axis II (SCID-II; [36]). Also, the use of a community sample and the limited experience of interviewers might confound the results, requiring replication studies on the clinical population with more experienced independent evaluators.

### Limitations and future direction

Despite the significance of introducing a Chinese version of STIPO to foster the dimensional diagnosis of PDs, this study has several limitations. First, while the present study aimed at examining the psychometric properties of STIPO-CH on a community sample and the clinical populations have been assessed, the small size and online recruitment might restrict its generalizability. The sample size also limited the use of more thorough statistical analyses, and not correcting the multiple correlations may inflate the chance of achieving statistical significance. Besides, only self-report measurements of personality pathology were administered as a criterion. These results could be inaccurate due to participants’ lack of self-awareness or social desirability bias. The fact that most interviewers were graduate students may also confound results because they lack insight into the concept of PO. Another shortcoming is that participants’ life functioning is not evaluated. Due to time limitation, no retest was taken following the initial interview. To this end, it is highly recommended that future researchers reexamine the psychometric properties of STIPO-CH with a bigger and more diverse sample that involves clinical patients. They should also carry out interviews that assess the level of PDs and general psychopathology with more experienced professionals and tracked the results in a longer term. Future studies should also conduct confirmatory factor analysis to evaluate the validity and collect information on life functioning to examine the external validity of STIPO-CH. It would be valuable to compare the structure and applicability between STIPO-CH and the newest STIPO-R.

It must also be admitted that the internal consistency of several dimensions was unacceptable. As this result may be attributed to cultural specificity, replication studies that explore the cultural sensitivity of STIPO-CH are considered highly beneficial. For example, as sex-relevant issues have long been taboo in China [37], participants may feel intense shyness and shame in answering relevant questions, report higher sexual inhibition, or have more difficulty experiencing sexual pleasure. Another frequently mentioned cross-cultural framework is the guilt emotion. Chinese participants may be more likely to mislabel the shame feelings as guilt since the latter is more allocentric and thus adheres to the collectivist cultural belief [38]. Taken together, such characteristics of the Chinese culture may cause distinct manifestations of borderline PO, requiring cross-cultural explorations of STIPO to enhance its cultural specificity and clinical utility.

## Conclusion

The Chinese version of the Structured Interview of Personality Organization (STIPO-CH) demonstrates acceptable reliability and validity as a potential instrument in clinical and research settings. As PO represents a dimensional model from a psychoanalytic perspective that receives increasing attention and acknowledgment, future research is recommended to investigate the psychometrics of STIPO-CH with various samples and criterion interviews to improve its generalizability and applicability.

## Data Availability

The data of this study are available upon reasonal request to the corresponding author.
